# Muscle fibre denervation in ageing

**DOI:** 10.1042/CS20260761

**Published:** 2026-06-10

**Authors:** Casper Soendenbroe

**Affiliations:** 1 The August Krogh Section for Human Physiology, Department of Nutrition, Exercise and Sports, University of Copenhagen, Copenhagen, Denmark; 2 Institute of Sports Medicine Copenhagen, Department of Orthopaedic Surgery, Copenhagen University Hospital - Bispebjerg and Frederiksberg, Copenhagen, Denmark

**Keywords:** aging, denervation, motor unit, neuromuscular junction, Sarcopenia, skeletal muscle

## Abstract

Muscle fibre denervation describes the loss of effective neural input from a motor neuron to one or more muscle fibres. In ageing, denervation is increasingly recognised as an important contributor to progressive declines in muscle strength and functional capacity, yet it remains heterogeneous and difficult to define in humans. This ambiguity reflects both biological complexity and current methodological limitations. The purpose of the present review is to synthesise current human evidence for muscle fibre denervation in ageing, clarify key conceptual distinctions, and evaluate methodological approaches used to assess denervation in humans. Muscle fibre denervation can occur through structural disconnection of the motor neuron from the fibre or through functional impairment of neuromuscular transmission. Evidence for denervation in ageing is derived from histological, molecular, electrophysiological, and circulating biomarker approaches, each capturing distinct and only partially overlapping aspects of neuromuscular integrity. Importantly, no single measure provides a comprehensive assessment of denervation. Experimental models of disuse in humans reveal a functional denervation phenotype, characterised by molecular and electrophysiological changes that partially resemble those observed with ageing. Physical activity appears to mitigate against aspects of muscle fibre denervation; however, the mechanisms underlying these effects remain incompletely understood. Collectively, the available evidence indicates that denervation in ageing is a multifaceted and dynamic process that requires multimodal, longitudinal approaches to define, detect, and ultimately target denervation-related mechanisms to preserve neuromuscular function across the human lifespan.

## Introduction

Each motor neuron and the muscle fibres it innervates form the functional units of the neuromuscular system (the motor unit), enabling voluntary movement and contributing to the maintenance of skeletal muscle mass, strength, and functional capacity [1]. Neuromuscular function can be disrupted by a wide range of insults, including pharmacological interventions, physical inactivity, ageing, and disease [2–4]. Among these, the ageing process has received increased attention, as accumulating evidence suggests that impaired neuromuscular integrity plays a central role in age-related declines in muscle mass and performance [5–7].

Recent methodological advances now enable more detailed assessment of neuromuscular structure and function in humans, including electrophysiological techniques, histological analyses, and molecular markers associated with denervation and reinnervation [8–12]. These advances have strengthened the link between ageing and loss of neuromuscular integrity but have also introduced ambiguity in how denervation is defined, detected, and interpreted. In particular, the term is increasingly used to describe phenomena that differ substantially in their underlying biology.

Addressing this conceptual tension requires a framework that captures both structural disconnection of the motor neuron from the muscle fibre and functional impairment of effective neural input. Evidence from histological, molecular, electrophysiological, and functional approaches must therefore be considered together, with the expectation that each capture overlapping but non-identical aspects of neuromuscular integrity. From this perspective, heterogeneity across methods should not be viewed as contradictory but as reflecting differences in the timing, severity, and duration of muscle fibre denervation. The overarching aim of the present review is to provide a coherent, human-centered synthesis for understanding muscle fibre denervation in ageing.

## What is meant by muscle fibre denervation?

The term denervation originates from 19th-century experimental neurophysiology, where it was used to describe the loss of neural input to a tissue, most commonly induced experimentally by nerve transection, excision, or ligation [13]. The word derives from Latin (*de*, meaning removal and *nervus*, meaning nerve), conveying its literal meaning. In clinical contexts, denervation has traditionally been associated with neurological disease and nerve trauma, where loss of motor neuron input to the muscle leads to rapid and profound muscle dysfunction [14].

**Figure 1:**
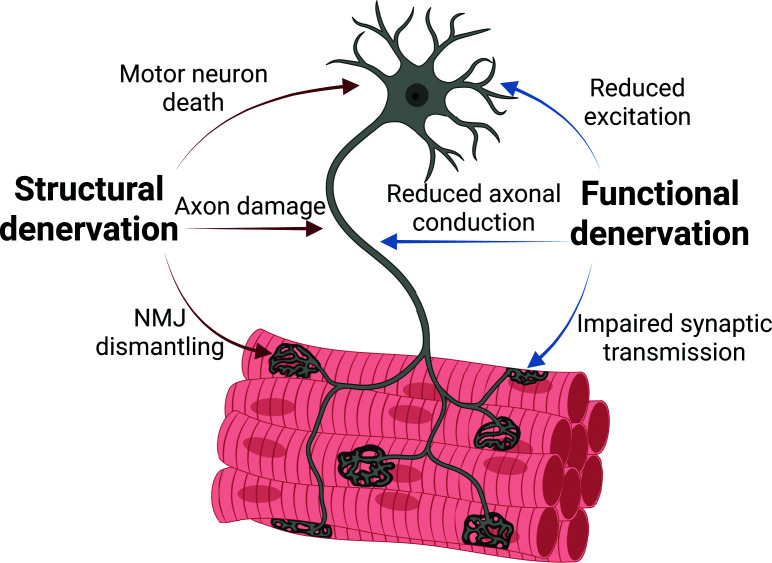
Structural and functional denervation in ageing skeletal muscle Schematic overview of structural and functional denervation in ageing skeletal muscle. Structural denervation involves loss of neural elements, including motor neuron death, axonal degeneration, and NMJ dismantling. Functional denervation reflects impaired neural transmission despite preserved anatomy, arising from reductions in firing, conduction, and synaptic efficacy. Functional denervation may precede irreversible structural loss and represents a potentially reversible stage of neuromuscular impairment.

In contemporary research, however, the term muscle fibre denervation is associated with considerable ambiguity, as it is applied to a range of structural and functional alterations that do not necessarily reflect complete loss of neural input (Figure 1). In the present review, structural denervation refers to anatomical disruption of the motor neuron-muscle fibre connection, whereas functional denervation refers to impaired effective neural activation of the muscle fibre despite a preserved structural connection. Classically, denervation referred to a strictly structural event, defined by loss of the motor neuron, physical disruption of the motor axon, or loss of effective pre- and postsynaptic contact at the neuromuscular junction (NMJ). Within this framework, evidence of denervation required direct anatomical or morphological evidence of nerve loss or NMJ disruption, demonstrating the removal of motor neuron input to the muscle fibre.

A broader interpretation emerges when *nerve* is understood as effective neural input rather than a physical structure. Under this interpretation, any process that prevents a motor neuron from successfully activating its target muscle fibres may be considered a form of functional denervation. Reductions in motor neuron excitation, impaired propagation of action potentials along the motor axon, or dysfunctional synaptic transmission at the NMJ each lead to diminished neural drive to the muscle fibre and induce molecular, electrophysiological, and structural alterations that closely resemble those observed following nerve transection [15,16]. These observations indicate that loss of neural input to the muscle fibre, rather than physical loss of the nerve or synapse itself, is sufficient to initiate several denervation-associated phenotypes.

**Figure 2:**
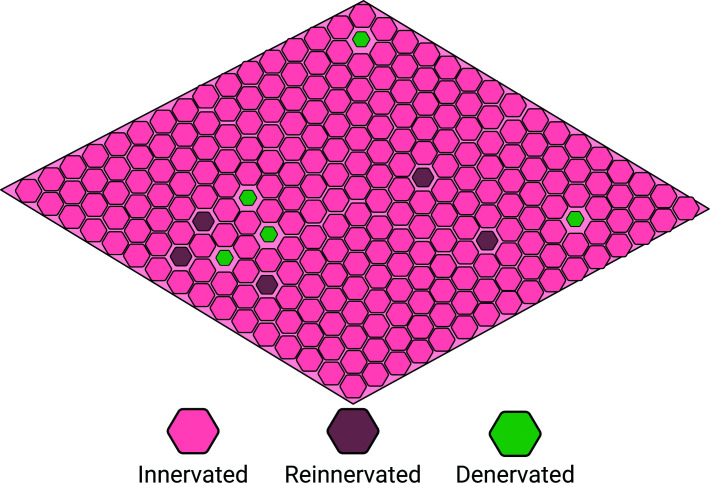
Dynamic mosaic of muscle fibre denervation and reinnervation in ageing Conceptual schematic of a whole muscle cross-section illustrating the spatial coexistence of normally innervated, denervated, and reinnervated fibres, reflecting ongoing cycles of denervation and compensatory reinnervation.

This conceptual distinction is particularly relevant in the context of ageing, where denervation of muscle fibres does not represent an acute, catastrophic event, as seen following nerve injury, but rather reflects a slow, progressive process unfolding over decades [17,18]. Given the thousands of motor neurons and the millions of muscle fibres they collectively innervate, it is likely that only a small fraction of fibres are affected at any given time (Figure 2) [19,20]. However, the cumulative consequence of this continuous turnover—characterised by repeated cycles of denervation and compensatory reinnervation—emerges as a central mechanism [21], contributing to age-related declines in muscle mass, strength, and physical function, ultimately predisposing to frailty, impaired mobility, and reduced quality of life [22,23].

Unless otherwise specified, the term denervation is used throughout the present review as an umbrella term encompassing both structural and functional denervation.This distinction is used as an operational framework, while recognising that available human methods often cannot distinguish these states directly.

## Evidence for muscle fibre denervation in ageing

It has long been recognised that physical damage to the nerve supplying a muscle leads to profound muscle atrophy [13]. Nerve transection in mice rapidly activates proteolytic pathways [24], shifting muscle protein balance towards net breakdown. These molecular changes translate into measurable muscle fibre atrophy within days to weeks [25,26], establishing denervation as one of the most potent catabolic stimuli for skeletal muscle. In ageing, however, denervation does not occur as an acute, global event affecting entire muscles. Instead, it reflects a slow, progressive, and spatially heterogeneous process, whereby a changing subset of muscle fibres undergoes denervation over time (Figure 2). As a consequence, individual muscles contain fibres reflecting distinct denervation events across both space and time, such that recently denervated, persistently denervated, actively reinnervating, and previously reinnervated fibres coexist within the same tissue. Collectively, these processes generate a dynamic mosaic of fibre states within ageing muscle. This spatial and temporal heterogeneity presents a major methodological challenge, as no single approach can adequately capture the full spectrum of denervation states within ageing muscle (Table 1).

**Table 1:** Modalities used to assess denervation in humans

#	Modality	What it measures	Strengths	Limitations
1	NMJ morphology	Structural synaptic integrity	Direct anatomical assessment	Rare, non-longitudinal, and very limited feasibility
2	Histology / IF	Fibre-level denervation markers and morphology	High specificity and spatial resolution	Sampling bias, low prevalence, and labour-intensive
3	Bulk molecular profiling	Denervation-related molecular programs in muscle tissue	High sensitivity, early detection, and established markers	Cellular averageing, non-muscle expression, and tissue requirement
4	Electrophysiology	Motor unit function and transmission	Functional relevance, *in vivo*	Low-force bias and indirect inference
5	Circulating biomarkers	Neural injury, axonal degeneration, and altered neuromuscular integrity	Scalable, minimally invasive, and longitudinal	Low anatomical specificity, high variability, and indirect

Overview of major methodological approaches used to assess muscle fibre denervation in human studies, highlighting their primary biological targets, strengths, and limitations.

### Direct assessment of the neuromuscular junction

In experimental animal models, direct assessment of NMJs is feasible. Immunofluorescence and imageing-based approaches allow detailed examination of pre- and postsynaptic structures, enabling classification of junctions as innervated, partially denervated, or denervated [27–31]. An expanding repertoire of markers and imageing modalities continues to refine how NMJ structure is assessed [32], and such approaches have provided clear temporal resolution of denervation processes with ageing in mice [28].

In humans, however, the situation is fundamentally different. Direct assessment of NMJs is largely limited to cadaveric or amputation material, which precludes longitudinal evaluation and often offers limited information regarding donor health status, physical activity, or neuromuscular history. Consequently, direct human evidence for age-related denervation at the NMJ is scarce. To date, only a small number of cross-sectional studies have directly examined the structure of human NMJs [32–42]. Among these, only three have explicitly evaluated age-related differences. Two studies report morphological alterations of the NMJ with ageing [40,42], including increased fragmentation and altered pre- and postsynaptic organisation, whereas a third reports that structural features of the human NMJ are preserved across the adult lifespan [38]. Together, these findings illustrate both the scarcity of direct human NMJ data and the lack of consensus regarding the extent to which NMJ structure is altered with ageing.

As a result of these constraints, evidence for muscle fibre denervation in ageing humans relies predominantly on indirect measures, including histological features of muscle tissue, molecular signatures associated with loss of neural input, electrophysiological assessments of motor unit function, and circulating biomarkers (Table 1).

### Histological and fibre level markers of denervation

**Figure 3:**
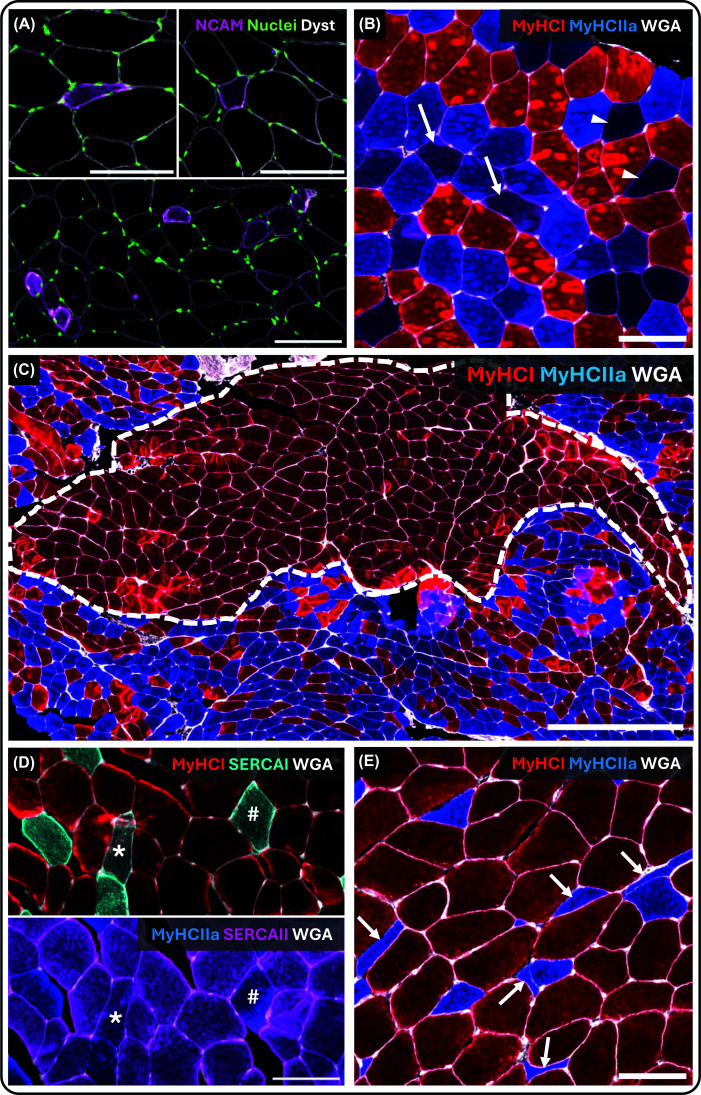
Histological indices of denervation in human skeletal muscle biopsies (**A**) NCAM-positive fibres, which are frequently atrophic and variably deformed, ranging from highly distorted morphologies (upper left) to more rounded profiles (upper right). Regions with increased local prevalence of NCAM-positive fibres are occasionally observed (bottom). NCAM (purple), nuclei (green), dystrophin (grey). Scale bar = 100 μm. (**B**) Hybrid fibres visualised by immunofluorescence staining for MyHC I (red), MyHC IIa (blue), and wheat germ agglutinin (WGA; grey). Purple fibres (arrows) indicate type I/IIa hybrids, whereas black or dark blue fibres (arrowheads) correspond to type IIa/IIx or IIx fibres. Scale bar = 100 μm. (**C**) Fibre-type grouping visualised using MyHC I (red), MyHC IIa (blue), and WGA (grey), illustrating a large fascicle composed almost exclusively of type I fibres adjacent to fascicles with mixed fibre-type composition. Grouped fibres are delineated by a white dotted line. Scale bar = 500 μm. (**D**) Serial sections illustrating fibre-type-specific expression of sarco/endoplasmic reticulum Ca^2+^-ATPase (SERCA) isoforms. Top: MyHC I (red), SERCA I (cyan), and WGA (grey). Bottom: MyHC IIa (blue), SERCA II (purple), and WGA (grey). Asterisks (*) indicate MyHC I-positive fibres co-expressing two SERCA isoforms, and hash symbols (#) indicate MyHC IIa-positive fibres co-expressing two SERCA isoforms. Scale bar = 100 μm. (**E**) Fibre deformity quantified using the shape factor index (SFI). Type II fibres exhibit marked atrophy and deformation, whereas type I fibres largely retain size and shape. Arrows indicate fibres with extreme levels of deformation (SFI >2.0). MyHC I (red), MyHC IIa (blue), and WGA (grey). Scale bar = 100 μm. Abbreviations: MyHC, myosin heavy chain; WGA, wheat germ agglutinin; SERCA, sarco/endoplasmic reticulum Ca^2+^-ATPase; NCAM, neural cell adhesion molecule; SFI, shape factor index.

Histological assessment of skeletal muscle tissue represents one of the primary approaches for inferring denervation in ageing humans. Chief among these is the analysis of gene and protein expression of skeletal muscle fibres, which can be readily obtained through muscle biopsies and applied in large human cohorts [9]. A wide range of genes and proteins have been linked to muscle fibre denervation and are therefore commonly used as markers to estimate its extent (see Figure 3 for examples). These include neural cell adhesion molecule (NCAM; Figure 3A), neonatal myosin heavy chain (MyHC), nestin, vimentin, denervation-associated sodium channel Nav_1_._5_, and several other proteins [43–45]. A shared feature of many such markers is the re-emergence of developmental expression programmes, consistent with loss of mature neural input [16]. Furthermore, denervation disrupts the normal activity-dependent restriction of acetylcholine receptor (AChR) expression to the postsynaptic membrane [46], resulting in increased extrasynaptic AChR expression detectable at both the transcript and protein levels [47]. Across several studies of healthy individuals, the absolute prevalence of fibres positive for various denervation-associated markers is typically low, often on the order of 0.0%–0.5% in young individuals and 0.5%–1.5% of fibres in older individuals [19,20,44,48–54], with somewhat higher proportions reported in even older populations [55]. Importantly, these estimates reflect only the fibres that are in a denervation-related state at the time of sampling. As such, even a low prevalence may translate into a substantial cumulative biological burden over the lifespan, given the slow, progressive, and spatially heterogeneous nature of age-related denervation.

This heterogeneity is also evident at the marker level, where different markers identify only partially overlapping fibre populations [19]. It is compounded by inconsistent intra-fibre staining patterns, whereby denervation-related proteins may be expressed in discrete regions rather than uniformly along the length or cross-section of a given fibre [16]. It should also be noted that some denervated fibres are markedly atrophic (cross-sectional area >2 SDs below the mean), such that their contribution to whole-muscle protein abundance is limited [20,56]. As a consequence, molecular signals originating from these fibres are likely to be substantially diluted in analyses based on whole-muscle homogenates, even if denervation contributes meaningfully to age-related muscle atrophy and functional decline. This underscores the value of fibre-level approaches over bulk tissue measurements. A further note of caution is warranted because several denervation-associated markers are not specific to denervation. For example, NCAM is expressed by myofibres at tissue junction sites [57]; developmental myosins are expressed by intrafusal fibres [58]; and several denervation-associated gene and protein markers are also induced during regeneration [59].

One classical histological approach to infer denervation and reinnervation is the quantification of grouped muscle fibres (Figure 3C). In healthy adult skeletal muscle, type I and type II fibres are typically arranged in a mosaic-like pattern, reflecting the intermingled distribution of fibres belonging to different motor units [60]. When muscle fibres become denervated, they may be reinnervated by collateral sprouts from surviving neighbouring motor axons [61,62]. If the reinnervating axon belongs to a motor unit with a different phenotype than the original motor neuron, the reinnervated fibres gradually acquire characteristics of the new motor unit, consistent with classic cross-innervation studies demonstrating neural regulation of muscle phenotype [63]. Repeated cycles of denervation and collateral reinnervation can, therefore, produce clusters of adjacent fibres expressing the same MyHC phenotype, commonly referred to as fibre type grouping [60]. Thus, fibre type grouping is generally interpreted as evidence of previous denervation followed by reinnervation, rather than as a marker of ongoing denervation [64].

Fibre type grouping has been analysed using several approaches, most notably one originally introduced by Jennekens et al. and a second later proposed by Kelly et al. [60,65]. A detailed methodological comparison is beyond the scope of the present review; readers are referred to recent work by Horwath and colleagues, who apply both approaches in parallel and discuss their respective strengths and limitations [49]. Importantly, both grouping metrics are inherently influenced by the underlying fibre type distribution and attempt to account for this in different ways. Nevertheless, muscles with a highly skewed fibre type composition will still exhibit a greater probability of forming larger or more numerous groups of the dominant fibre type, with the converse applying to less abundant fibre types. Interpretation of fibre type grouping is therefore sensitive to fibre type composition.

A further complication arises from growing evidence that substantial heterogeneity exists within traditional fibre type classifications, such that fibres categorised as the same type may differ in molecular, metabolic, and functional properties [66]. This intra-fibre type heterogeneity challenges the assumption that grouped fibres necessarily represent homogeneous reinnervated units and is discussed further in a later section on mismatches in protein expression. Despite these limitations, a number of studies have reported a greater proportion of grouped fibres in muscles of older compared with younger individuals [20,49,54,65,67], whereas others have not observed such differences [68,69], likely reflecting methodological variation across studies.

Although increased fibre type grouping is commonly interpreted as evidence of denervation followed by collateral reinnervation [5], this interpretation depends on knowing what happened to the total pool of muscle fibres. A grouped pattern could arise because denervated fibres were successfully reinnervated by neighbouring motor axons and subsequently changed fibre type. However, a similar pattern could also arise if denervated fibres failed to be reinnervated and were instead lost, leaving the remaining fibres distributed in a more grouped pattern. These scenarios reflect different biological outcomes but may appear similar in a biopsy section. In small animal muscles, total fibre number can be determined from whole-muscle sections [70], making it possible to assess whether fibre loss has occurred. This is not possible in human biopsy studies, where a biopsy captures only a small fraction of the muscle, often ranging from a few hundred to a few thousand fibres, compared with several hundred thousand fibres in the vastus lateralis alone [17]. Thus, in human biopsies, fibre type grouping should be interpreted with caution.

Denervation has also been associated with mismatches in protein expression within individual muscle fibres. One well-described example is the presence of hybrid fibres, defined by co-expression of multiple MyHC isoforms within the same fibre [71] (Figure 3B), though not necessarily within the same region of the fibre [72]. The prevalence of hybrid fibres is influenced by neural input [73], physical activity [74], disuse [75] and ageing [76], and has been examined using a range of methodological approaches, including single-fibre analyses, muscle homogenates, and cross-sectional histology. In cross-sectional analyses, hybrid fibres are commonly identified using immunofluorescence, sometimes relying on staining for a single MyHC isoform [44], with hybrids inferred from weak staining. While this approach captures some degree of MyHC co-expression, it represents only a limited subset of the broader spectrum, as hybrid fibres can occur between all fibre types and across a continuum of relative myosin expression levels [66].

Historically, myofibrillar ATPase histochemistry has provided superior resolution of relative MyHC composition within individual fibres, whereas immunofluorescence offers greater practicality and throughput but primarily yields qualitative information on co-expression [77]. Consequently, immunofluorescence-based assessments are well suited for identifying the presence of hybrid fibres, but less informative regarding the relative contribution of individual MyHC isoforms within a given fibre. Beyond MyHCs, neural input regulates a broad range of muscle proteins, and denervation may therefore result in mismatched expression of additional fibre type-specific proteins [78]. Supporting this notion, studies have reported mismatches between contractile and calcium-handling proteins, such as SERCA isoforms, and the nominal fibre type [65], suggesting that altered neural input can uncouple coordinated protein expression programmes within muscle fibres (Figure 3D). Given the large number of proteins regulated in a fibre type-dependent manner, additional mismatches likely exist but remain underexplored. Because proteins differ substantially in turnover rates, for instance, in relation to resistance exercise [12], some mismatched expression patterns may reflect residual signatures of prior innervation states rather than current fibre identity. In this view, such patterns can be interpreted as vestiges of muscle fibre type switching, capturing a history of altered neural input rather than a uniform denervated state.

More recently, muscle fibre deformity has emerged as a potential additional discriminator of denervation [79] (Figure 3E). In human skeletal muscle, most fibres exhibit relatively uniform shape characteristics, reflected by the SFI (fibre perimeter^2^/4 × π × fibre cross-sectional area), with typical values ranging from ∼1.2–1.4 and slightly higher in type II compared with type I fibres. In older individuals, particularly individuals beyond the eighth decade of life, mean SFI values are higher, especially in type II fibres (∼1.6), and a subset of fibres display pronounced deformity (>1.8). Importantly, fibre deformity does not uniformly accompany the expression of denervation-associated markers such as NCAM. While NCAM-positive fibres are occasionally highly deformed, many retain a relatively preserved geometry, indicating that denervation *per se* is not sufficient to induce morphological deformation.

Skeletal muscle is a densely packed tissue in which individual fibres are subjected to continuous mechanical constraints imposed by surrounding fibres and connective tissue [80,81]. Under normal conditions, regular fibre activation and force generation contribute to the maintenance of a stable, near-polygonal (penta- or hexagonal) fibre shape [80]. Fibres that are structurally or functionally denervated lose this stabilising influence, rendering them more susceptible to passive deformation through compression by neighbouring, actively contracting fibres [82]. NCAM-positive fibres exhibiting marked deformity may represent fibres that are unable to generate sufficient intrinsic force to resist externally imposed compressive loads, whereas NCAM-positive fibres with a more preserved geometry may reflect fibres undergoing successful or ongoing reinnervation.

A parallel can be drawn to observations in other, simplified, cellular systems, in which mechanical forces within tissues act as a selective pressure, favouring cells that are better able to transmit and withstand fluctuating mechanical loads [83]. In such systems, mechanically robust cells tend to remain stably integrated, whereas mechanically weaker cells undergo progressive deformation and, in some contexts, eventual elimination [84]. In skeletal muscle, fibres are embedded within a complex connective tissue network and transmit forces both longitudinally and laterally via costameres [85]. Denervated fibres generate little or no force yet continue to experience compressive loads from surrounding, contracting fibres [82]. Consequently, even when metabolically viable, such fibres may become mechanically disadvantaged within the tissue. This framework provides a plausible explanation for the pronounced deformity observed in some denervated fibres.

Whether mechanically imposed constraints also contribute to the loss of these fibres during ageing remains unknown, but the concept offers a unifying mechanical interpretation linking denervation markers to altered fibre geometry. SFI has been shown to differ between muscles in both humans and rodents [81], likely reflecting differences in muscle architecture and loading patterns [86,87]. However, whether fibre deformity is similarly induced by other physiological or experimental perturbations, such as disuse, remains unknown. In ageing, only a small fraction of fibres is affected by denervation at any given time, generating a mechanically asymmetric force environment that may predispose selectively affected fibres to compression and deformation. In contrast, experimental disuse imposes a more uniform reduction in activation across fibres, resulting in largely symmetric unloading of the tissue and little rationale for increased compressive stress on individual fibres. Together, these considerations suggest that fibre deformity may be specific to conditions characterised by heterogeneous neural withdrawal rather than generalised unloading.

### Molecular signatures associated with denervation

While histological approaches provide spatially resolved information on muscle fibre structure and protein expression, they may be limited in their ability to detect early or subtle changes in neural input. Molecular analyses based on quantitative PCR and bulk RNA sequencing offer a complementary perspective by capturing transcriptional responses of muscle tissue to altered neural activation [25]. These approaches are highly sensitive and can detect denervation-associated gene programmes that may precede histological changes, with a small number of genes repeatedly linked to denervation or reduced neural activation [9,88]. These include the AChRs (CHRNA1, CHRNB1, CHRNG, CHRNE, and CHRNG), which show higher expression in older compared with younger individuals [44,53]. Additional denervation-associated genes include NCAM1, NES (Nestin), RUNX1, AGRN (Agrin), MUSK, and other developmentally regulated transcripts that are re-expressed in muscle fibres following loss of neural input [89].

Importantly, inference of denervation from bulk gene expression data remains indirect. Transcriptomic analyses performed on whole muscle homogenates reflect an average signal across multiple fibre types, connective tissue, and non-muscle cell populations. Moreover, some commonly used denervation-associated transcripts are not exclusive to muscle fibres but are also expressed by other cell types within muscle tissue, such as NCAM (also known as CD56) in satellite cells [90], nestin in proliferating endothelial cells [91], and vimentin in fibroblasts [45]. When gene expression analyses are combined with parallel histological evaluation, major structural features—such as NMJs, myotendinous junctions, and connective tissue content—can be partially accounted for [45,53,56]. Nevertheless, several confounding processes may produce transcriptional signatures that overlap with denervation, including muscle regeneration and muscle pathology [59,92], both of which activate gene programmes similar to those induced by loss of neural input.

Despite these interpretational challenges, the physiological relevance of denervation-associated transcriptional signatures has only recently begun to be addressed in the context of human ageing. While the detrimental effects of acute denervation on muscle mass, physical function, and overall health are well established in experimental animal models and in clinical settings involving nerve injury or neuromuscular disease [14,25], direct links between molecular indices of denervation and functional outcomes in ageing humans have been limited. Recently, however, a large, well-phenotyped cohort study demonstrated that higher expression of denervation-responsive gene programmes is associated with lower muscle volume and poorer functional outcomes, including reduced strength and aerobic capacity [9]. Such integrative analyses, combining high-quality molecular profiling with detailed *in vivo* physiological phenotyping, are essential to establish the functional significance of molecular markers of denervation in ageing humans.

Despite emerging functional associations, bulk transcriptomic approaches remain limited in resolving the cellular origin and spatial context of denervation-associated signals. This limitation has motivated the rapid expansion of single-cell and single-nucleus transcriptomic approaches in skeletal muscle. Owing to the multinucleated nature of muscle fibres, conventional single-cell RNA sequencing preferentially captures mononuclear cell populations, such as endothelial cells, satellite cells, fibroblasts, and immune cells, while largely excluding myonuclei [8]. In contrast, single-nucleus RNA sequencing enables transcriptional profiling of myonuclei in addition to non-muscle cell types [8], making the two approaches complementary. Because denervation represents a profound perturbation of the neuromuscular system, it is likely to influence multiple cell populations within muscle tissue [93]. Nevertheless, a primary focus of the present review is on the transcriptional changes within the myonuclear compartment, as these nuclei directly integrate neural input [94]. Accumulating evidence indicates that myonuclei within a single multinucleated fibre are transcriptionally heterogeneous and spatially specialised [95]. In particular, a subset of myonuclei located near the NMJ—commonly referred to as NMJ-associated myonuclei—has been identified [96–98], alongside transcriptionally distinct populations associated with the myotendinous junction [95,97].

NMJ-associated myonuclei are enriched for transcripts involved in synaptic organisation and maintenance and play a key role in sustaining effective neuromuscular transmission [97]. Following denervation, NMJ-associated myonuclei show down-regulation of innervation-related genes, whereas cell body myonuclei—by far the most abundant population—exhibit induction of denervation-responsive gene programs. This compartmentalised response highlights that denervation does not elicit a uniform transcriptional program across the muscle fibre. Building on these observations, recent transcriptomic studies have further delineated myonuclear responses to sciatic nerve transection, identifying gene programs that are restricted to specific myonuclear subsets and therefore obscured in bulk tissue analyses [97,99]. Collectively, these findings demonstrate that neural input is sensed and regulated at the level of individual myonuclei in a transcriptionally heterogeneous manner.

To date, however, most of these mechanistic insights derive from rodent models, and their translation to human ageing remains an open challenge. Large single-nucleus atlases of human skeletal muscle have begun to map age-related transcriptional changes across myonuclei and non-muscle cell populations [8,100]. However, their ability to resolve denervation-related processes is currently constrained by several factors. Denervated fibres in healthy humans constitute only a small fraction of the total fibre pool at any given time, requiring substantial sequencing depth to reliably capture affected myonuclei [101]. This challenge is compounded by the likelihood that denervated fibres exist across different stages of denervation and reinnervation, each associated with distinct transcriptional states [25]. As a result, denervation-related signals may be diluted within large atlases optimised to capture broad features of ageing rather than rare, transient neuromuscular events. These limitations complicate interpretation of single-nucleus datasets in relation to overall denervation burden at the tissue level and underscore the continued need for hypothesis-driven studies of neuromuscular integrity.

### Electrophysiological evidence of altered neural input

Electrophysiological approaches provide functional readouts of neural input to skeletal muscle and represent the longest-standing methods for studying neuromuscular function [13]. Unlike histological or transcriptomic approaches, electrophysiological methods assess signal transmission and motor unit behaviour *in vivo*, thereby capturing the functional consequences of altered neural input. If structure governs function, a degree of correspondence between structural alterations of the neuromuscular system and electrophysiological readouts would be expected. Indeed, such correspondence is observed in some [31], but not all [102,103], studies.

The main methodological approach is electromyography (EMG). Traditionally, surface EMG has been used to characterise global muscle activation patterns, whereas intramuscular EMG enables assessment of individual motor unit behaviour [104]. More recently, high-density surface EMG has emerged as a powerful approach that allows decomposition of motor unit discharge properties during voluntary contractions [105]. Each of these techniques offers distinct advantages and limitations with respect to invasiveness, spatial resolution, and interpretability. Although longitudinal electrophysiological assessments are feasible, repeated measurements are methodologically demanding, particularly due to sensitivity to electrode placement and sampling variability. Longitudinal electrophysiology is feasible, but methodologically demanding, and interpretation must account for sampling variability and technical noise [106]. A number of comprehensive reviews have addressed electrophysiological adaptations of the neuromuscular system with ageing and inactivity [3,4]. In the present review, the focus is restricted to electrophysiological measures most directly relevant to altered neural input and denervation-related processes in humans, including motor unit number estimates (MUNE), motor unit action potential stability, motor unit firing behaviour, and persistent inward currents.

MUNE describes an array of methods that provides an indirect estimate of the number of functional motor units innervating a muscle by dividing the compound muscle action potential elicited by supramaximal nerve stimulation by the average single motor unit potential, yielding a non-invasive index of electrically active motor units [107]. The technique has been used for several decades across multiple human muscles and consistently demonstrates age-related reductions in estimated motor unit number [108]. Electrophysiological indices of NMJ transmission stability, derived from intramuscular EMG, provide functional measures of synaptic transmission stability [109]. These measures are used in clinical neurophysiology [110] but have been applied relatively infrequently in studies of normal human ageing, where the limited available evidence points to modest age-related alterations across older adults with differing levels of physical function [10,111,112]. Across two of these studies, older adults exhibited greater variability in neuromuscular transmission [10,111], which has been interpreted as reflecting increased instability at the NMJ within individual motor units [109]. Importantly, these assessments were obtained during low-level voluntary contractions (≈10%–25% of maximal voluntary contraction), such that larger, higher-threshold motor units—which are not recruited under these conditions—may exhibit distinct alterations that are not captured by these measurements.

Beyond indices of synaptic transmission stability, growing attention has been directed toward age-related changes in persistent inward currents (PICs) within motor neurons. PICs are intrinsic motoneuron conductances that amplify synaptic input and sustain motor unit firing, thereby enhancing force output for a given level of descending neural drive [113]. Recent studies indicate that PIC estimates are reduced with ageing and muscle disuse, as reviewed elsewhere [114]. Because PICs play a central role in transforming synaptic input into effective motor output, their attenuation provides a plausible neural mechanism contributing to the disproportionate decline in muscle strength relative to muscle mass observed with ageing.

### Circulating biomarkers of neuromuscular disturbance

Circulating biomarkers offer an attractive, non-invasive approach to assess neuromuscular disturbance in humans. Their main advantages include minimal burden to participants, feasibility in large cohorts, and the ability to obtain repeated measurements with high temporal resolution. In addition, most circulating markers are relatively inexpensive and technically straightforward to implement. However, these advantages are offset by significant limitations. Circulating biomarkers generally exhibit low anatomical specificity, raise questions regarding tissue origin [115], and, in most cases, lack validation against more direct assessments of neuromuscular structure or function [116]. As such, they should be interpreted with caution.

Among the many proposed candidates, the most extensively studied biomarkers in relation to neuromuscular integrity are C-terminal agrin fragment (CAF) and neurofilament light chain (NF-L) [117]. CAF is a cleavage product of agrin, a nerve-derived trophic factor essential for the formation and maintenance of the NMJ, generated through neurotrypsin-mediated processing at the NMJ [118,119]. Weak to moderate associations have been reported between circulating CAF levels and age as well as measures of physical function [120], whereas CAF concentrations increase in response to short-term disuse interventions [50,52] and decrease following exercise interventions in some [121–123], but not all studies [116,124,125]. Despite this, direct associations between circulating CAF levels and established tissue-based markers of denervation in humans remain limited. Moreover, agrin is expressed in multiple tissues, including high levels in kidneys [115], and circulating CAF levels are affected by pharmacological interventions such as antihypertensive treatment [125], indicating that its biological interpretation remains incomplete.

NF-L is a structural neuronal cytoskeletal protein released following axonal damage and is widely used as a biomarker of neurodegeneration [126]. Circulating NF-L levels increase with ageing [127] and show sensitivity to periods of physical inactivity, including short-term limb immobilisation [52]. These findings suggest that NF-L may capture aspects of neuronal vulnerability associated with disuse and ageing. However, NF-L lacks specificity for the neuromuscular system, and it remains unclear whether observed increases reflect degeneration of central neurons, peripheral motor axons, or a combination of both. A number of other circulating biomarkers have also been associated with physical function but generally lack specificity for neuromuscular changes. Growth differentiation factor-15, for example, is linked to physical frailty [128], yet its relationship to denervation appears indirect and likely reflects broader systemic stress rather than alterations in neural input to muscle per se [129].

Together, circulating biomarkers provide scalable indicators of neuromuscular disturbance but currently lack the anatomical specificity required to serve as direct measures of muscle fibre denervation. As with most blood-based biomarkers, their interpretation is further complicated by diurnal variation [130], sensitivity to hydration status [131], which is frequently altered in older and frail individuals, and biological variability related to acute physiological stress and metabolic state. Accordingly, their greatest utility is likely to lie in complementing tissue-based, electrophysiological, and functional assessments, where they may improve phenotyping and risk stratification rather than serve as standalone diagnostic tools. Circulating biomarkers of neuromuscular and neural integrity, including CAF and NF-L, have been reviewed in more detail elsewhere, with emphasis on their responsiveness to ageing, disease, physical activity, and disuse [117,126,132,133].

## Factors and insults influencing denervation

Muscle fibre denervation in ageing is not an isolated process but rather reflects the cumulative influence of external and internal modifiers acting on the neuromuscular system across the lifespan. Neural input to skeletal muscle is shaped by behavioural and mechanical factors, most prominently physical activity and its absence, including periods of disuse. This section therefore focuses on common and clinically relevant modifiers for which human evidence exists.

### Physical activity

Physical activity is widely regarded as a protective factor for neuromuscular health, although the mechanisms by which it influences muscle fibre innervation remain incompletely understood [134]. Using both cross-sectional and longitudinal study designs, muscle strength has consistently been shown to decline more rapidly with ageing than muscle mass [135,136]. This dissociation is commonly interpreted as indirect evidence of neuromuscular involvement in the ageing process, as functional losses exceed those expected from changes in muscle size alone. However, this interpretation is not specific to denervation, as age-related changes in muscle quality, excitation-contraction coupling, tendon properties, and force transmission may also contribute to the disproportionate loss of strength [137].

Importantly, comparisons between physically active and inactive older adults demonstrate greater preservation of both muscle mass and strength in active individuals [44,138,139]. Moreover, short-term intervention studies in older adults show that skeletal muscle retains a substantial capacity to adapt to increased mechanical loading [59,140–143] indicating that a substantial component of age-related neuromuscular decline reflects modifiable processes rather than irreversible structural loss.

Yet, despite these clearly beneficial effects, the mechanisms by which physical activity influences neuromuscular ageing remain uncertain. To address this gap, it is necessary to consider where within the neuromuscular system exercise-induced adaptations may arise. One possibility is that physical activity acts at the level of the motor neuron itself, by preserving neuronal viability or slowing age-related loss. Several lines of evidence suggest that the number of motor units, neurons and axons is reduced in older adults [18,108,144], raising the possibility that activity-dependent signalling influences neuronal survival. Such effects could, in principle, be mediated by muscle-derived factors capable of modulating motor neuron health. Behavioural cues such as physical activity can be imprinted onto cells, allowing experimental interrogation of exercise-associated signalling *in vitro* [44,145–147], while acknowledging the simplified nature of these models [148]. Using this approach, a recent study demonstrated that cells isolated from lifelong exercisers exerted protective effects on motor neurons *in vitro*, providing experimental evidence in a reductionist model that long-term physical activity can induce neuroprotective properties in muscle-derived cells [11]. Importantly, these effects were observed over short experimental durations and required direct contact between myotubes (i.e., primitive muscle fibres) and motor neurons, a configuration that differs markedly from the *in vivo* situation, where motor neuron cell bodies are separated from muscle fibres by long axonal projections.

This anatomical separation necessitates long-range communication, of which retrograde signalling provides a biologically plausible pathway for such effects. This process has been demonstrated experimentally [149,150] and is also exploited *in vivo* by certain neurotropic toxins to gain access to the central nervous system [151]. Although direct evidence that physical activity confers neuroprotection via retrograde signalling is currently lacking, negative effects conferred throughout this pathway have been implicated in disease contexts, including experimental evidence that retrograde transport can exacerbate disease progression in amyotrophic lateral sclerosis [152].

An alternative, non-mutually exclusive mechanism is the secretion of muscle-derived factors into the circulation. Skeletal muscle releases a wide array of molecules, as demonstrated by proteomic analyses of conditioned media [153], and several of these factors are known to cross the blood-brain barrier [154]. This notion is supported by plasma transfer studies showing that exercise-induced circulating factors can lower neuroinflammation and improve brain function [155]. However, the majority of this evidence derives from animal models, and whether comparable circulating mechanisms contribute to motor neuron or NMJ maintenance in ageing humans remains uncertain. More broadly, longitudinal studies in older adults have shown that declines in muscle strength and contractile function track with changes in brain structure and cognition, supporting the concept that neuromuscular and central nervous system ageing are also coupled in humans [156–158].

Alternatively, physical activity may exert its primary effects at the level of the NMJ, enhancing synaptic stability or transmission efficacy without necessarily preventing motor neuron loss. A substantial body of animal literature supports activity-dependent maintenance of NMJ structure and function, including preservation of postsynaptic organisation and reduced synaptic fragmentation [30], as reviewed elsewhere [159]. In humans, where direct structural assessment of NMJs is limited, exercise has nonetheless been shown to modulate molecular features of the postsynaptic apparatus, including AChR gene expression [53]. Such molecular adaptations occur over time scales far shorter than those required for changes in motor neuron number, supporting a direct effect of exercise on neuromuscular transmission. By contrast, relatively few studies have examined the effects of longer-term training interventions on NMJ-related markers in humans, and findings are mixed. Some markers show training-associated improvements, whereas others remain unchanged, making it difficult to draw firm conclusions [51,56]. Cross-sectional evidence, however, suggests differences between physically active and sedentary individuals [20,44,54,160].

From an electrophysiological perspective, the picture remains debated [161,162]. While it is unclear whether physical activity preserves motor unit number with ageing [163,164], evidence exists that motor units are larger in physically active individuals (i.e., each motor neuron controlling more muscle fibres) [165], consistent with an enhanced capacity for reinnervation. Such findings suggest that exercise does not necessarily prevent denervation but may facilitate compensatory expansion of surviving motor units. This interpretation is consistent with earlier observations in rodents demonstrating collateral nerve sprouting following denervation, accompanied by increases in motor unit size and remodelling of motor unit territories [61,62].

Together, these findings suggest that the strongest evidence currently supports a role for physical activity in promoting compensatory reinnervation, although contributions from preservation of motor neurons cannot be excluded. Distinguishing between preservation of motor neurons and enhancement of compensatory reinnervation remains a central challenge in understanding how physical activity modulates ageing of the neuromuscular system.

### Disuse and physical inactivity

A number of clinical conditions, including chronic obstructive pulmonary disease [166], critical illness [167], and sepsis [168], are associated with features suggestive of impaired neuromuscular integrity, such as disproportionate losses of muscle strength, altered motor unit function, and molecular markers linked to denervation. However, in these settings, disease-specific factors—such as systemic inflammation, metabolic stress, hypoxia, and pharmacological treatment—are intertwined with varying degrees of physical inactivity. As a result, the relative contribution of disease processes versus reduced neural activation to denervation-related phenotypes cannot be readily disentangled in clinical populations.

To address this limitation, experimental models of disuse—including bed rest, limb unloading, limb immobilisation, and dry immersion—have been used to isolate the effects of reduced mechanical loading and neural activation in otherwise healthy individuals. These models allow direct examination of the neuromuscular consequences of disuse in the absence of confounding disease. Accordingly, a growing body of evidence from such experimental studies demonstrates that disuse is accompanied by a denervation-like phenotype [39,48,50,52,169,170].

Such changes have been detected across molecular, electrophysiological, and circulating biomarker measures [50,52,170]. For example, Arentson-Lantz and colleagues demonstrated an increased number of NCAM-positive muscle fibres following disuse in humans [169], findings that have since been replicated by others [48,52]. One of the most consistent molecular responses to disuse is an increase in AChR gene expression, most commonly involving the α- and δ-subunits, as demonstrated across several human disuse studies [52,171,172]. These transcripts also rank among the most robustly up-regulated genes in meta-analyses of disuse-induced muscle remodelling [15]. In this context, preliminary observations from two cohorts (*n* = 68) undergoing short-term limb immobilisation indicate a >6-fold increase in expression of selected AChR subunits.

Beyond transcriptional changes, disuse is accompanied by consistent alterations in neuromuscular function. Electrophysiological studies show that unloading or immobilisation increases motor unit action potential complexity [52], reduces motor unit firing rates [170], and alters discharge behaviour [173], collectively indicating impaired neural drive rather than loss of contractile capacity alone. More recently, disuse has also been shown to reduce estimates of persistent inward currents in motor neurons [174]. Because persistent inwards currents amplify synaptic input and sustain motor neuron firing [113], their reduction provides a plausible neural mechanism linking disuse to the disproportionate loss of muscle force relative to muscle mass [175,176], a dissociation that closely mirrors that observed with ageing.

Taken together, these findings suggest that many functional consequences attributed to denervation may arise from impaired NMJ transmission rather than complete structural nerve-muscle disconnection. In this context, functional denervation may precede, and in some cases occur independently of, structural denervation. A useful illustration of this principle comes from NMJ development, where synaptic stability is strongly activity-dependent, and inactive or weakly transmitting synapses are progressively pruned without loss of the parent motor neuron [177]. Extending this analogy, functional denervation may represent an early and potentially reversible stage in which neural input is insufficient to sustain normal signalling but complete structural disconnection has not yet occurred. During this stage, neuromuscular transmission may still be restored, allowing the muscle fibre to remain viable; however, the duration for which such a state can be maintained and the conditions under which recovery remains possible are currently unknown.

## Methodological challenges for assessing muscle fibre denervation

Assessing muscle fibre denervation in humans presents substantial methodological challenges, largely reflecting the indirect nature of most available approaches. Direct structural assessment of NMJs is typically limited to post-mortem or surgical material and cannot be applied longitudinally. Consequently, most human studies rely on indirect markers, each capturing only selected aspects of neuromuscular integrity.

A central challenge arising from this reliance is the pronounced heterogeneity of denervation-associated signatures [10,19,49,66,97]. No single gene, protein, or functional measure uniformly identifies all denervated fibres; instead, different markers label partially overlapping subsets. This heterogeneity likely reflects differences in the timing, severity, and duration of muscle fibre denervation, as well as the dynamic balance between denervation and reinnervation, thereby limiting the interpretability of any single marker when used in isolation.

Morphological variability further complicates interpretation. Fibres expressing denervation-associated markers span a wide range of sizes and shapes [79], and several commonly used markers are also expressed in contexts unrelated to denervation, including myotendinous junctions, muscle spindles, and regeneration following injury [45,59]. Additional complexity arises from the generally low prevalence of denervated fibres in humans, which renders repeated biopsy sampling inherently variable. Even when biopsies are carefully standardised for depth and orientation and randomised for position along the muscle, longitudinal sampling remains subject to considerable biological noise, which may obscure subtle changes over time. This was clearly illustrated in a control group undergoing repeated biopsies over 16 weeks without any intervention, where substantial intra-individual variability was observed [56]. If denervated fibres are assumed to be stochastically distributed within the tissue, such variability is expected to generate random noise rather than systematic bias. Nevertheless, overcoming this noise requires careful anatomical standardisation, sufficiently large sample sizes, and rigorous analytical strategies to enable detection of meaningful longitudinal effects.

Electrophysiological approaches introduce additional constraints, as many techniques rely on low-force contractions that preferentially sample small, low-threshold motor units [170]. Alterations affecting larger, high-threshold motor units, often implicated in ageing, may therefore be under-represented, potentially contributing to discrepancies between electrophysiological and tissue-based findings.

Taken together, these limitations argue against single-marker approaches to denervation assessment. Instead, the most informative strategy is likely a multimodal one, integrating complementary histological, molecular, electrophysiological, and, where relevant, circulating measures. Such integration enables denervation-related heterogeneity to be interpreted as biologically meaningful rather than methodological noise and may provide a more robust estimate of overall denervation burden than any individual readout alone.

## Future perspectives

Future progress in understanding muscle fibre denervation with ageing in humans will require large, longitudinal, multimodal studies capable of resolving when denervation emerges, how it evolves over time, and whether it is reversible. To date, most human evidence derives from cross-sectional [19,49] or short-term intervention studies [52,56], which have been valuable for identifying candidate markers but are inherently limited in their ability to capture denervation dynamics over longer time periods (i.e., years).

Further advances will require multimodal studies that integrate molecular, histological, electrophysiological, and functional measures within the same experimental designs. Progress will also depend on linking processes across biological scales. Recent ultrastructural studies reveal highly interconnected myofibrillar networks, whose organisation varies with muscle type, fibre type, maturation, and possibly ageing [178,179]. This parallels evidence that muscle fibre morphology is shaped by muscle type, fibre type, exercise, and ageing [49,79,81], and extends upward to differences in whole-muscle architecture and geometry [180,181]. Together, these findings suggest that denervation-related perturbations may be encoded across biological scales of muscle organisation, from myofibrillar architecture to whole-muscle structure.

Progress in this area will also depend on continued methodological development. While no single technique is likely to provide a definitive measure of muscle fibre denervation, emerging approaches may help place denervation-related changes into a broader structural and functional context. Advances in quantitative and diffusion-based magnetic resonance imaging allow increasingly detailed, non-invasive assessment of muscle architecture in humans [180], potentially providing a link between fibre-level denervation phenotypes and whole-muscle structure *in vivo* [182]. In parallel, improved understanding of mechanical forces within muscle fibres and myofibrillar networks offers a basis for interpreting how altered activation shapes muscle morphology over time [84,178]. Electrophysiological methods are also evolving, with improved analytical pipelines and emerging intramuscular high-density EMG techniques enabling more detailed and longitudinal assessment of neuromuscular transmission [183,184]. At the molecular level, omics-based approaches—including transcriptomics, proteomics, and metabolomics—provide a more comprehensive view of denervation-related changes than targeted molecular analyses [185]. When combined with robust, longitudinal study designs, these approaches offer the opportunity to uncover coordinated molecular adaptations to altered neural input that would not be detectable using predefined candidate markers alone. Ultimately, the key challenge will not be the availability of methods, but the integration and validation of these approaches within coherent, longitudinal study designs.

Finally, exercise remains the most effective and best-established strategy for preserving neuromuscular function [134], yet important questions remain regarding mechanisms of action, the minimal effective dose, and inter-individual responsiveness [143]. Pharmacological strategies may provide complementary support in selected populations [186], but progress in this area has been slow, and such approaches should be viewed as adjuncts rather than alternatives to physical activity. In the longer term, improved molecular characterisation of denervated muscle fibres may open avenues for more selective, targeted interventions, though substantial conceptual and technical challenges remain.

## Conclusions

In conclusion, the present review synthesises current human evidence for muscle fibre denervation in ageing, highlighting both the diversity of methods used to assess neuromuscular integrity and the resulting heterogeneity in reported findings. Collectively, the available data indicate that denervation-related phenotypes are a prominent feature of ageing skeletal muscle but are expressed in a complex and multifaceted manner that reflects both biological variability and methodological limitations.

## Open Access

Open access for this article was enabled by the participation of the University of Copenhagen in an all-inclusive Read & Publish agreement with Portland Press and the Biochemical Society under an agreement with the Royal Danish Library.

## Abbreviations

CAF: C-terminal agrin fragment
EMG: electromyography
MyHC: myosin heavy chain
NCAM: Neural cell adhesion molecule
NF-L: neurofilament light chain
NMJ: neuromuscular junction
PICs: persistent inward currents
SFI: shape factor index
